# Identification of DHX9 as a cell cycle regulated nucleolar recruitment factor for CIZ1

**DOI:** 10.1038/s41598-020-75160-z

**Published:** 2020-10-22

**Authors:** Urvi Thacker, Tekle Pauzaite, James Tollitt, Maria Twardowska, Charlotte Harrison, Adam Dowle, Dawn Coverley, Nikki A. Copeland

**Affiliations:** 1 Biomedical and Life Sciences, Faculty of Health and Medicine, University of Lancaster, Lancaster, LA1 4YQ UK; 2 Department of Biology, University of York, Heslington, York, YO10 5DD UK; 3 Metabolomics and Proteomics Laboratory, York Bioscience Technology Facility, University of York, Heslington, York, YO10 5DD UK

**Keywords:** Biochemistry, Cell biology, Molecular biology

## Abstract

CIP1-interacting zinc finger protein 1 (CIZ1) is a nuclear matrix associated protein that facilitates a number of nuclear functions including initiation of DNA replication, epigenetic maintenance and associates with the inactive X-chromosome. Here, to gain more insight into the protein networks that underpin this diverse functionality, molecular panning and mass spectrometry are used to identify protein interaction partners of CIZ1, and CIZ1 replication domain (CIZ1-RD). STRING analysis of CIZ1 interaction partners identified 2 functional clusters: ribosomal subunits and nucleolar proteins including the DEAD box helicases, DHX9, DDX5 and DDX17. DHX9 shares common functions with CIZ1, including interaction with XIST long-non-coding RNA, epigenetic maintenance and regulation of DNA replication. Functional characterisation of the CIZ1-DHX9 complex showed that CIZ1-DHX9 interact in vitro and dynamically colocalise within the nucleolus from early to mid S-phase. CIZ1-DHX9 nucleolar colocalisation is dependent upon RNA polymerase I activity and is abolished by depletion of DHX9. In addition, depletion of DHX9 reduced cell cycle progression from G1 to S-phase in mouse fibroblasts. The data suggest that DHX9-CIZ1 are required for efficient cell cycle progression at the G1/S transition and that nucleolar recruitment is integral to their mechanism of action.

## Introduction

The precise duplication of the genome is a highly regulated process that ensures genomic stability. The formation of putative replication origins, origin licensing and initiation of DNA replication are highly orchestrated processes that are tightly regulated by sequential cyclin-CDK complexes and Dbf4 dependent kinase (DDK)^1,2^. DNA replication initiates at thousands of replication origins to facilitate efficient and expedient duplication of the genome^3,4^. Temporospatial regulation of DNA replication is mediated by recruitment of cyclin dependent kinases to chromatin, a process that is mediated in part by Cip1 interacting zinc finger protein 1 (CIZ1)^5^. CIZ1 promotes DNA replication in mammalian cells^6^ and is part of an extraction-resistant compartment, or nuclear matrix, in the nucleus^7^. CIZ1 interacts with pre-replication complex proteins Cdc6^8^, cell cycle regulators cyclin E, cyclin A^5^ and the CDK inhibitor protein p21^9^ consistent with a role in cell cycle regulation^10^.

**Figure 1:**
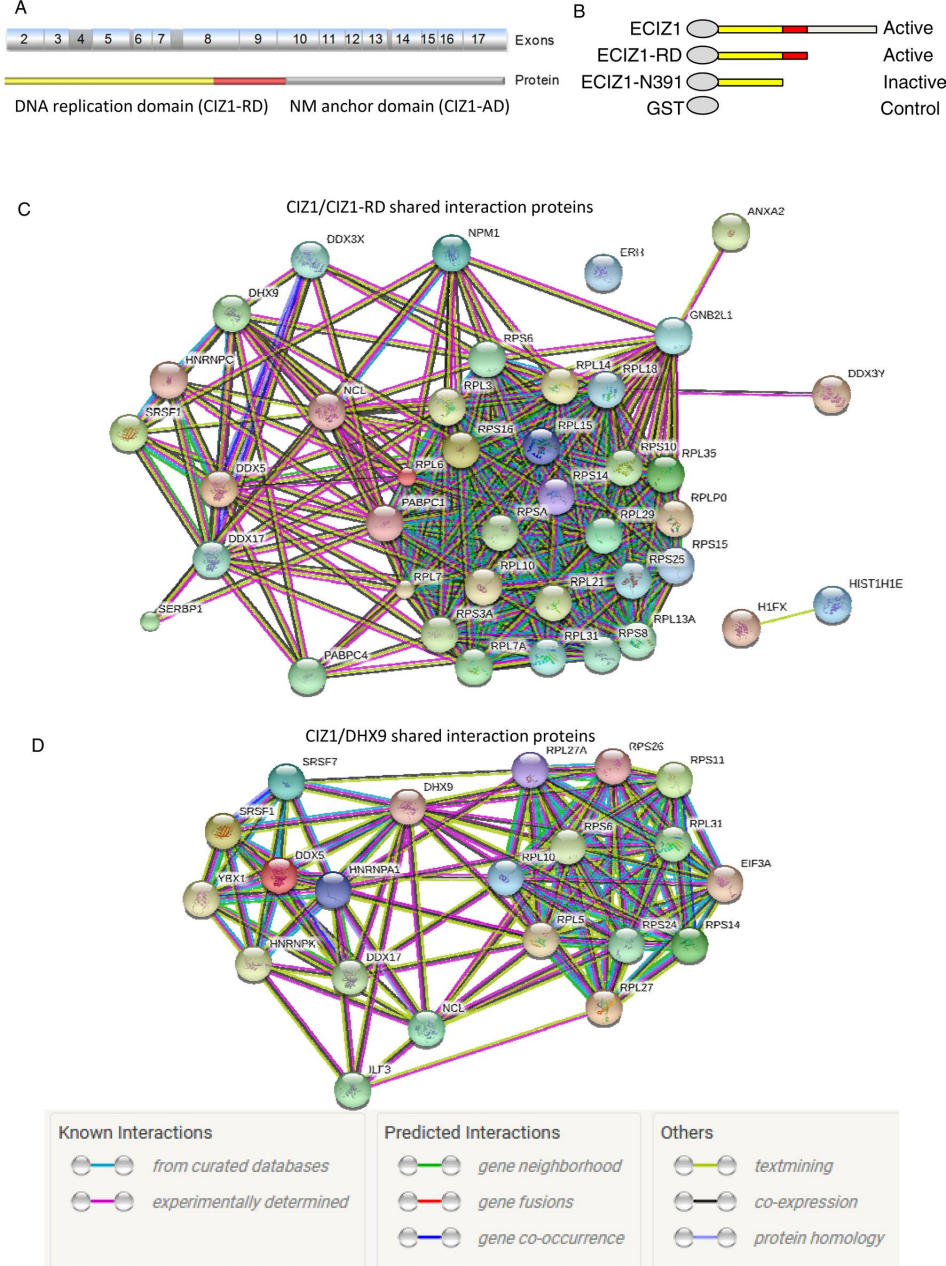
Identification of CIZ1 interaction partners by MALDI MS/MS. (**A**) Schematic of murine CIZ1, showing translated exons (numbered), and those that encode the DNA replication domain encoded by CIZ1 fragment RD (exons 2–9 and part of 10, shown in yellow and red box)^5^. The nuclear matrix anchor domain-containing fragment (exon 10–17; grey box) is also shown^7^. Reported CIZ1 spicing events^16,17,37,69^ are shaded in dark grey in exons 4, 6, 8, and 14. (**B**) Four constructs were prepared for interaction screen: GST-ECIZ1, GST-ECIZ1 RD and DNA replication inactive GST-ECIZ1 N391. GST was used as a negative control. (**C**) MALDI-MS/MS and mascot database searching was used to identify interaction partners for CIZ1 (Table S1–4). STRING analysis^68^ of ECIZ1 and ECIZ1-RD shared Interaction partners identify two clusters: RNA binding/helicases (left) and ribosomal cluster (right). Functional interaction data is shown in key. (**D**) Common CIZ1-DHX9 interaction partners were analysed by STRING analysis. The proteins identified here share RNA binding/helicase activities and ribosomal subunits as seen for CIZ1.

CIZ1 can be divided into two functional domains. The N-terminal replication domain (CIZ1-RD, also termed CIZ1-N471 in earlier work) associates with cyclin A to promote initiation of DNA replication in vitro and the C-terminal anchor domain (CIZ1-AD) localises CIZ1 to the DNase-resistant nuclear matrix (Fig. 1A)^5,7^. CIZ1 mediates recruitment of cyclin A to non-chromatin structures^5^. CIZ1 DNA replication activity is mediated through cyclin A interactions as mutation of the Cy ii-motif (K/RXL, K321) prevented cyclin A binding and abolished CIZ1’s DNA replication activity^5^. CDK-mediated phosphorylation of CIZ1 at multiple sites also regulates cyclin interaction and CIZ1 activity^8^. CIZ1 promotes cyclin A recruitment and initiation of DNA replication in a hypophosphorylated state, while hyperphosphorylation abolishes CIZ1 DNA replication activity. This describes an autoregulatory feedback loop that may contribute to mechanisms that ensure replication occurs exactly once per cell cycle^8^.

CIZ1 contributes several processes in addition to its role in cyclin recruitment to the nuclear matrix. CIZ1 interacts with the inactive X-chromosome in female cells^11–13^, is implicated in oestrogen receptor mediated transcriptional regulation^14^. Mutation of the *CIZ1* gene has been linked with varied diseases including cervical dystonia^15^ and Alzheimer’s disease^16^. Furthermore, CIZ1 overexpression or mis-splicing promotes tumour cell growth and targeted depletion of CIZ1 can reduce cellular proliferation or tumour size^10,17–23^. In addition, CIZ1 also displays tumour suppressor activity in CIZ1 ablated murine models^11,24^. Both deletion and overexpression of CIZ1 induces delocalisation of the non-coding X-inactive specific transcript (XIST) RNA from the inactive X chromosome (Xi)^12^, suggesting that the levels of CIZ1 are important in the regulation of multiple processes.

To gain a more detailed understanding of CIZ1 function, we exploited characterised CIZ1 constructs that are capable of promoting initiation of DNA replication in vitro*,* to identify interaction partners in soluble extracts from HeLa cells. This approach identified the DEAH box helicase DHX9 as the most significant interaction partner and the focus of this work is to functionally characterise their interaction. DHX9 is an RNA/DNA helicase associated with many aspects of nucleic acid metabolism including DNA repair^25^, pre-mRNA splicing^26,27^, RNA interference^28^, translation^29^ and DNA replication^25,30,31^. Importantly, DHX9 shares common functions with CIZ1, including interaction with XIST long-non-coding RNA^11,12,32^, epigenetic regulation^13,33^ and regulation of DNA replication^5,8,34^. DHX9 has been implicated in the DNA replication process through interaction with DNA polymerases α, δ, and ε^25^, PCNA^30^, DNA topoisomerase II^31^ and binding to active DNA replication origins^34^. DHX9 associates with filamentous actin present at the nucleolar periphery^35^, which is the site for ribosomal DNA (rDNA) replication^36^. Finally, DHX9 localises to the nucleolus during differentiation in embryonic stem cells and regulates heterochromatin formation^33^. The data presented here identify nuclear proteins that associate with CIZ1 that will enable further characterisation of CIZ1 function with emphasis on in vitro and in vivo characterisation of the CIZ1-DHX9 complex. This work suggests DHX9 is required for Ciz1 nucleolar recruitment, and is required for efficient cell cycle progression at the G1/S transition.

## Results

### Identification of CIZ1 interaction partners during S-phase

The CIZ1 variant, ECIZ1 was cloned from an embryonic murine cDNA library and lacks the polyglutamine N-terminal sequences and is readily purified as recombinant ECIZ1 from *E. coli*^6,7^. Three functionally characterised murine ECIZ1-derived constructs were used to generate GST-tagged recombinant proteins (Fig. 1A,B) to profile interaction partners in soluble extracts from HeLa cells. This approach has been used previously to identify and analyse cyclin A-CDK2^5^ and Cdc6 interactions^8^. Both ECIZ1^5,6,8^ and ECIZ1-derived ECIZ1-RD^5,8^ encode proteins that support initiation of DNA replication, while derived truncated fragment (ECIZ1-N391^5,37^) does not support DNA replication^5^. All three baits, and GST alone, were incubated with nuclear extracts prepared from HeLa cells synchronised in early S-phase by double thymidine arrest. Recovered proteins were identified after on-bead trypsin digestion and liquid chromatography tandem mass spectrometry (LC MS/MS) using Mascot database searching, and significant hits selected with confidence limits ≥ 99.5%, ≥ 2 peptides and best ion CI ≥ 99%. This yielded 53, 107, 2 and 1 interactors for ECIZ1, ECIZ1-RD, ECIZ1-N391 and GST respectively (Table S1–4). Due to the low number of hits for control (GST) and DNA replication-inactive truncate N391, the analysis here focussed on DNA replication-competent ECIZ1 and ECIZ1-RD.

To provide greater insight into the biological significance of the CIZ1, CIZ1-RD dataset the interaction proteins were analysed using STRING^38^. This approach groups Ciz1-interacting proteins by functional relationships generating two clusters for both CIZ1 and CIZ1-RD (Figure S1,S2), ribosomal subunits (ECIZ1 False detection Rate (FDR) = 6e^−48^ Table S1, CIZ-RD FDR = 2.3e^−82^ Table S2) and DEAD box/ribonucleoprotein complexes (ECIZ1 FDR = 2.3e^−51^, CIZ1-RD FDR = 1.3e^−40^). Moreover, comparison of the interaction partner lists revealed that 42/53 ECIZ1 interaction partners were common to CIZ1-RD, with 9/10 present in both CIZ1 and CIZ-RD interaction partners (Table 1; Table S5; Fig. 1C). Significantly, only CIZ1 constructs that promote cell cycle progression (ECIZ1 and ECIZ1-RD), but not inactive CIZ1-N391 construct, were able to interact with these complexes. CIZ1 has been shown to regulate cell cycle progression^5,8^ and regulate localisation of XIST^11,12^. Therefore, it is striking that DDX5, DDX17 and DHX9 were identified in the top 10 hits for both CIZ1 and CIZ1-RD (Table 1) are XIST interaction partners^32^ and that DHX9 has also been reported to regulate cell cycle progression^34^.

**Table 1:** The top 10 interaction partners by confidence limits for CIZ1 and CIZ1-RD.

Rank	ECIZ1 interactors	Total on score	CIZ1-RD interactors	Total on score
1	DHX9	579	DHX9	1514
2	Hist1h1e	517	ANXA2	967
3	ANXA2	376	DDX17	669
4	RPL15	344	RPL6	625
5	DDX17	337	DDX5	537
6	DDX5	257	DDX21	496
7	RPS10	230	RPS10	457
8	RPS3a	206	RPL15	454
9	RPS8	195	GNB2L1	447
10	CBR1	181	NPM1	437

Proteins highlighted in bold were found to interact with both CIZ1 and CIZ1-RD.

DHX9 (also known as RNA helicase A and NDH II, Uniprot number: Q08211) was the top hit for both CIZ1 and CIZ1-RD. DHX9 is an RNA and DNA helicase that is proposed to function in several processes, including activation of DNA replication origins^30^, translational efficiency^35^, RNA interference^36^, viral infection^37^ and interacts with XIST RNA^11,12^. Of the known DHX9 interactor proteins in Biogrid^38^, 21/239 are also identified in our ECIZ1 or CIZ1-RD interactors. Analysis of CIZ1-DHX9 interaction partners in STRING^38^ returned GO terms ribosome biogenesis and RNA metabolism with highest significance functions of the CIZ1 interactors (Fig. 1D; Table S6).

### CIZ1 and DHX9 form a stable complex in vitro

**Figure 2:**
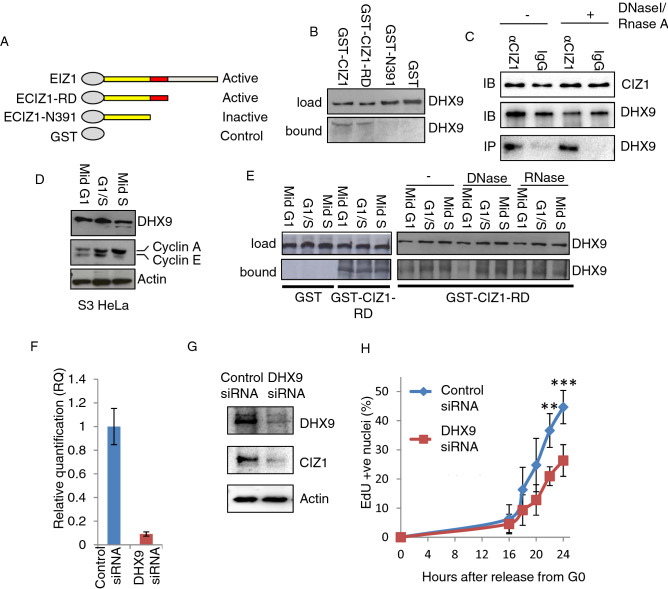
CIZ1 directly associates with DHX9 in vitro. (**A**) Schematic representation of four constructs for interaction screen: GST-ECIZ1, GST-ECIZ1-RD, GST-ECIZ1 N391 and GST was used as a negative control. (**B**) Products of GST-CIZ1 interaction assay showing DHX9 detected by western blot. Upper panel shows load proteins, lower panel recovered DHX9. (**C**) Immunoprecipitation (IP) reactions from S-phase HeLa nuclear extracts. Pre-immune IgG controls and anti-CIZ1 N471 antibody and RNAse A/DNase I were added as indicated. Upper panel shows CIZ1 and DHX9 load. Lower panel detected DHX9 after IP. (**D**) Synchronised G1, G1/S and Mid S-phase HeLa S3 extracts were prepared and synchronisation confirmed by cyclin E and A abundance^5^. (**E**) CIZ1-DHX9 interaction in mid G1, Late G1 or S-phase extracts using the indicated constructs. Left panel, GST control and RD; right panel, CIZ1-RD ± DNase or RNase added to binding reactions as indicated. (**F**) Quantitative reverse transcriptase PCR (qRT-PCR) analysis of DHX9 transcripts, 24 h after transfection of mouse NIH3T3 cells with control siRNA and DHX9 siRNA. Transcript levels for DHX9 are shown relative to GAPDH. G) Western blots showing effect of control siRNA and DHX9 siRNA on DHX9, CIZ1 and actin. Full-length blots are found in supplemental material. (**H**) Mouse NIH3T3 cells were synchronised in G0, siRNA treated and cell cycle re-entry monitored by EdU incorporation. The percentage of cells in S-phase for control (blue) and DHX9 depleted cells (red) are shown. Statistical significance was determined by ANOVA plot with Tukey post hoc analysis for time points are shown. **indicates *P* < 0.01 and ***indicates *P* < 0.001.

To validate the CIZ1-DHX9 interaction, GST-ECIZ1 and derived fragments (Fig. 2A) were incubated with S-phase HeLa nuclear extracts and bound proteins analysed by western blot. Consistent with mass spectrometry results both ECIZ1 and CIZ1-RD recovered DHX9 (Fig. 2B, Figure S3) while N391 did not, highlighting a dependence on the 80 amino-acid sequence previously shown to be required for CIZ1 DNA replication activity^5^. Next to assess if CIZ1 and DHX9 associate in vivo, immunoprecipitation assays were performed using matched IgG from pre-immune rabbits and CIZ1 specific antibodies (Fig. 2C). This demonstrated that endogenous CIZ1 and DHX9 associate in vivo and this is independent of DNA and RNA. To assess whether there was a cell-cycle dependence to this interaction, nuclear extracts were isolated from HeLa cells synchronised in G1 phase, early or mid S-phase. Synchronisation was confirmed by expression of cyclin E and A as cyclin E peaks in late G1 phase, then decreases as cyclin A accumulates during S-phase entry^5^ (Fig. 2D, Figure S3). DHX9 was expressed at similar levels in all three extracts and was successfully retrieved by GST-CIZ1, and GST-CIZ1-RD, indicating a stable interaction mediated via the N-terminal domain of CIZ1 (Fig. 2E, left panel; Figure S3). As CIZ1 and DHX9 can interact with nucleic acids^39–41^, binding assays were performed with DNase 1 or RNase A. In this context, CIZ1 and CIZ1-RD were able to recover DHX9 from nuclear extracts (Fig. 2E, right panel), although there was reduced recovery with DNase 1 in mid G1 extracts that may be associated with a reduced affinity in G1 phase that increases in S-phase (Fig. 2E, Figure S3). These data show that CIZ1-DHX9 interact throughout G1 and S-phase, and suggest that this interaction is not dependent on nucleic acids.

To assess whether DHX9 has a role in regulation of the cell cycle, mouse fibroblasts were synchronised in G0 by contact inhibition and serum starvation^5,6,8,37,42^ and transfected with control siRNA or anti-DHX9 siRNAs followed by release into the cell cycle. This resulted in a reduction in both DHX9 transcript (Fig. 2F) and protein levels (Fig. 2G, Figure S3). There is also a reduction in CIZ1 levels that may be due reduced cell cycle progression, as CIZ1 levels accumulate from the G1/S transition^8^. In addition, we cannot exclude that possibility that DHX9 increases CIZ1 stability preventing its degradation in this phase of the cell cycle. To determine if DHX9 depletion affects cell cycle re-entry was monitored during the following 24 h by EdU incorporation to determine the proportion of cells in S-phase. Depletion of DHX9 was associated with a significant decrease relative to controls at 22 and 24 h (Fig. 2H). These observations parallel similar analysis of the effect of depletion of CIZ1, which also resulted in restrained entry to S-phase, via a mechanism that interferes with timely pre-initiation complex (pre-IC) conversion^5,6^. The reduction in CIZ1 protein levels, caused by depletion of DHX9, may be associated with a reduction in S-phase cells (Fig. 2H), as CIZ1 expression increases during G1 phase and is maximally expressed in S-phase^8^. Alternatively, this could be more directly related to suppression of DHX9-containing protein complexes. Nevertheless, the data presented here are consistent with other analyses that demonstrated that DHX9 is required for efficient cell cycle progression and initiation of DNA replication^34^.

### DHX9-CIZ1 dynamically co-localise in the nucleolus in early S-phase

**Figure 3:**
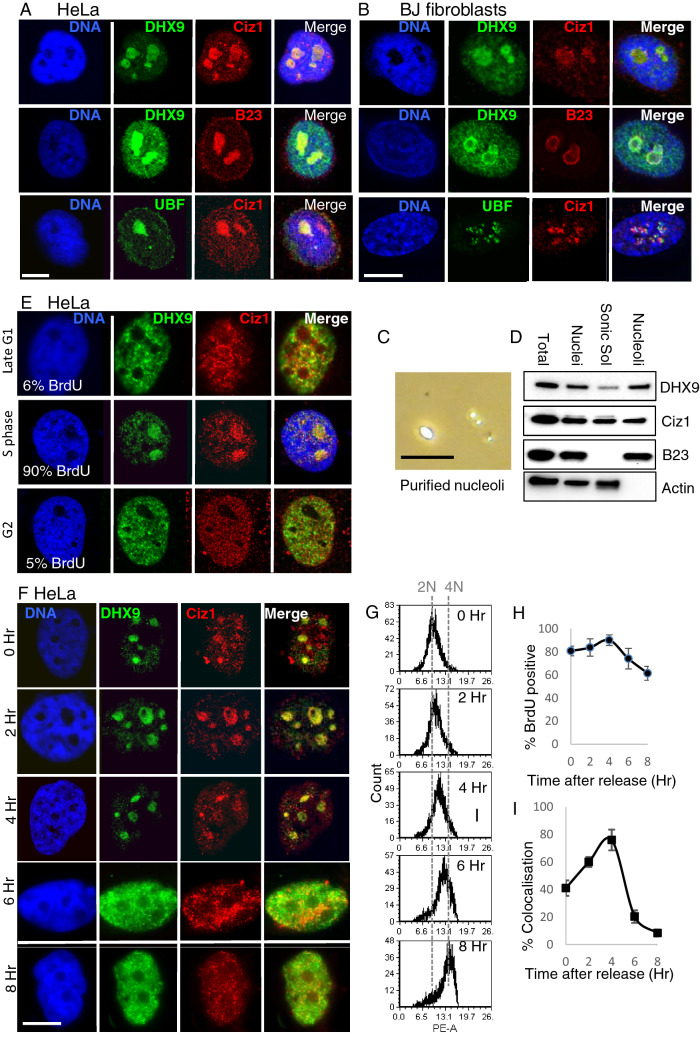
CIZ1 colocalises with DHX9 in the nucleolus of cells normal fibroblasts and HeLa cells. (**A**) HeLa cells were imaged by immunofluorescence confocal microscopy. Upper panel: Total DNA is shown in blue; DHX9, green; CIZ1, Red and merged image. Middle panel: Total DNA shown in blue; DHX9, green; nucleophosmin (B23), Red and merged image. Lower panel: Total DNA shown in blue; UBF, green; CIZ1, Red and merged image. Yellow indicates colocalisation. White bar = 10 µm (**B**) As for A except human BJ fibroblasts were used. (**C**) Differential interference contrast light microscopy showing purified nucleoli. Black bar = 10 µm. (**D**) Western blot analysis of nucleoli from (**C**) for DHX9, CIZ1, nucleophosmin (B23) and actin. (**E**) Representative HeLa cell nuclei imaged by immunofluorescence confocal microscopy after synchronisation in G1, S and G2 phase. Percentage of cells in S-phase in each population, as determined by BrdU incorporation, is shown. Total DNA is stained with DAPI (blue), CIZ1 (red) and DHX9 (green). White scale bar = 10 µm. (**F**) HeLa cells were synchronised in early S-phase with thymidine treatment and CIZ1 and DHX9 visualised by confocal fluorescence microscopy. Representative images were taken as indicated after removal of thymidine. Total DNA is shown in blue; DHX9, green; CIZ1, red and merged image, with yellow showing colocalisation. (**G**) Flow cytometry profiles of propidium iodide stained DNA in HeLa cells at indicated time points after release from thymidine block. (**H**) Percentage of BrdU positive S-phase cells determined by pulse labelling at indicated time points after release from thymidine block. Data shows mean ± SD, where 100 nuclei scored for each time point I) Percentage of nuclei showing nucleolar colocalisation for CIZ1/DHX9 at indicated time points. Data from three independent experiments showing mean ± SD, where > 50 nuclei scored for each time point.

DHX9 localises to the nucleolus in mouse fibroblasts^43^, but CIZ1 is not known to accumulate at the nucleolus. However, nucleolar localisation is a common feature of ECIZ1 binding proteins as identified by STRING analysis with 19/53 for ECIZ1 (FDR = 3.5e^−12^) and 36/107 for CIZ1-RD (FDR = 7.26e^−23^) (Table S1, S2). Immunofluorescence revealed that CIZ1 and DHX9 colocalised in regions with low DAPI fluorescence, consistent with the nucleolus (Fig. 3A, Figure S4S4/Supplementary video 1 and Figure S5A). Next, the nucleolar markers nucleophosmin (B23) and upstream binding factor (UBF) were used to determine if DHX9 and CIZ1 were localised within the nucleolus. Immunofluorescence confocal microscopy was used with nucleolar markers nucleophosmin (B23) in combination with DHX9, and upstream binding factor (UBF) in combination with CIZ1 (Fig. 3A). The data indicate that CIZ1 is localised to the nucleolus in a subset of cells, where it was found to be colocalised with DHX9 or UBF, suggesting that CIZ1 is present in both the fibrillar center (FC) and granular component (GC). The data presented are consistent with a nucleolar function for CIZ1 and DHX9.

CIZ1 is associated with the inactive X-chromosome in normal female cells, visible as a condensed heterochromatic region within the nucleus^11–13^ typically proximal to the nucleolus or nuclear envelope. In addition, in primary cells Ciz1 is transiently associated with perinucleolar regions^13^. To confirm that the CIZ1 aggregates shown here are independent of the inactive X-chromosome, the hTERT-immortalized human male foreskin fibroblast BJ cell line was used. CIZ1-DHX9 was visualised in the nucleolus, yielding consistent results to HeLa cells (Fig. 3B). In BJ cells, CIZ1-UBF and DHX9-nucleophosmin show nucleolar colocalisation (Fig. 3B). Thus, nucleolar CIZ1-DHX9 is found in both male and female human cell lines. To further support the nucleolar localisation of CIZ1 and DHX9, nuclei were purified by sequential sucrose step fractionation and sonication^44^. Nucleoli were purified from HeLa cells and this showed that DHX9 and CIZ1 were present in the nuclear and nucleolar fractions. DHX9 was not detected in the nucleosolic fraction after sonication, whereas CIZ1 was detected consistent with the release of non-nucleolar CIZ1 (Fig. 3C, D, and Figure S6).

To investigate whether nucleolar localisation of CIZ1 and DHX9 are cell cycle regulated, HeLa cells were synchronised by two methods: mitotic release using nocodazole (Figure S4), and double thymidine arrest and release (Fig. 3). Both synchronisation approaches revealed that CIZ1-DHX9 were enriched at nucleoli in early to mid S-phase (Fig. 3E; Figure S5A). Representative images at two-hour intervals after release from the thymidine block are shown (Fig. 3F), and cell cycle progression confirmed by flow cytometry (Fig. 3G) and S-phase labelling with BrdU (Fig. 3H). Quantification of CIZ1-DHX9 nucleolar localisation through S-phase (Fig. 3I) confirmed that CIZ1-DHX9 peaks 4 h after release from thymidine block, which reduced sharply as cells progressed into late S-phase (6–8 h after release; Fig. 3I; Figure S5B). Importantly, similar results were found for a population of HeLa cells synchronised arrested in mitosis using nocodazole (Figure S7), suggesting this response is not due to the drug induced cell cycle synchronisation. This analysis of the temporal dynamics of CIZ1-DHX9 colocalisation points to an S-phase specific function and suggests that CIZ1 dynamically associates with nucleoli in human cell lines.

### CIZ1 nucleolar localisation is DHX9 dependent

**Figure 4:**
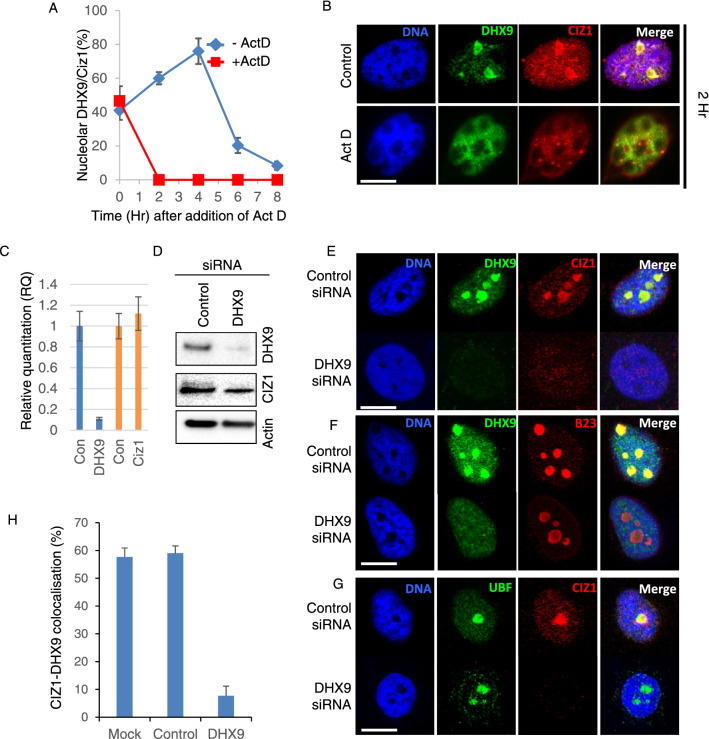
CIZ1 localisation to the nucleolus requires DHX9. (**A**) Percentage of cells showing nucleolar localisation of CIZ1-DHX9 as determined by confocal microscopy. For triplicate experiments showing mean ± SD, where > 50 nuclei were scored for each time point. (**B**) Confocal immunofluorescence microscopy showing total DNA is shown in blue; DHX9, green; CIZ1, Red and merged image, with yellow showing colocalization, ± Actinomycin D. (**C**) Relative quantification of DHX9 transcript levels for control and DHX9 siRNA treated cells 24 h post-transfection. (**D**) Western blot analysis of DHX9 depleted cells showing levels of DHX9, CIZ1, nucleophosmin and actin protein. (**E**) Confocal fluorescence microscopy of HeLa cells showing CIZ1/DHX9 after control siRNA or DHX9 siRNA transfection. Total DNA is shown in blue; DHX9, green; CIZ1, Red and merged image, with yellow showing colocalisation. (**F**) Nucleolar localisation of nucleophosmin (B23) is independent of DHX9 expression. Total DNA is shown in blue; DHX9, green; nucleophosmin (B23), Red and merged image, with yellow showing colocalisation. (**G**) Nucleolar localisation of UBF is independent of DHX9 expression. Total DNA is shown in blue; UBF, green; CIZ1, red and merged image, with yellow showing colocalisation. (**H**) Percentage of cells showing nucleolar localisation of CIZ1-DHX9 as determined by confocal microscopy data shows mean ± SD, from 3 independent experiments each with > 50 nuclei scored.

The interaction between CIZ1 and DHX9 in vitro, and their nucleolar colocalisation during early S-phase suggests a common function. To determine whether CIZ1 nucleolar localisation is dependent on DHX9, DHX9 nucleolar localisation was disrupted by addition of nanomolar quantities of actinomycin D that inhibits RNA polymerase I and abolishes DHX9 nucleolar localisation^43^. HeLa cells were treated with actinomycin D, and the distribution of CIZ1 and DHX9 determined using immunofluorescence microscopy. Quantification of the number of cells with colocalised nucleolar CIZ1-DHX9 showed reduced DHX9 nucleolar localisation with a concomitant reduction in CIZ1 nucleolar localisation (Fig. 4 A, B; Figure S5B). This suggests that DHX9 may facilitate recruitment of CIZ1 to the nucleolus. Next, to assess the whether DHX9 is required for CIZ1 nucleolar localisation, DHX9 siRNA were used to deplete DHX9 in asynchronous HeLa cells. This resulted in efficient depletion of DHX9 transcript (Fig. 4C) and protein levels (Fig. 4D; Figure S8). Monitoring the nucleolar CIZ1 levels in control siRNA and mock depleted cells showed ~ 60% of cells displayed colocalised nucleolar CIZ1 and DHX9 (Fig. 4E–H). This contrasts with DHX9 depleted nuclei, where CIZ1 is mostly not detected in the nucleolus, with only ~ 8% of cells showing nucleolar CIZ1-DHX9 (Fig. 4E, H; Figure S9). DHX9 depletion does not affect nucleolar localisation of B23 (Fig. 4F; Figure S10) and UBF (Fig. 4G) indicating a functional, intact nucleolus. Thus, both siRNA mediated depletion of DHX9, and RNA polymerase I inhibition promotes nucleolar exclusion of DHX9 and CIZ1. Taken together these data suggest that DHX9 is required for CIZ1 nucleolar recruitment to maintain efficient cell cycle progression.

## Discussion

This work has identified a non-biased interaction network for CIZ1 using synchronised S-phase nucleosolic proteins. CIZ1 associates with DEAD/H Box helicases, splicing factors, regulators of ribosomal function, known XIST interactors and ribonucleoprotein complexes. The CIZ1 interactor proteins identified here show a significant enrichment of nucleolar factors for both CIZ1 and CIZ1-RD (Table S5; FDR = 4.2e^−12^), consistent with the cell cycle regulated nucleolar location that we observe in early to mid S-phase HeLa cells. However, our analysis did not recover cyclin subunits, which are known to interact with CIZ1 and are required for its DNA replication activity. We demonstrated previously that the cyclin A-CIZ1 interaction is sensitive to CDK-mediated phosphorylation at three sites (T144, T192, T293), which significantly weakens their affinity^8^. This is most likely because the conditions used here utilised S-phase extracts with physiological ATP levels, which is known to reduce CIZ1-cyclin affinity in vitro due to CDK mediated phosphorylation of CIZ1^5,8^.

The most significant hit was DHX9, a RNA and DNA helicase that has been associated with activation of DNA replication origins^34^, translational efficiency^45^, RNA interference^46^, viral infection^47^, regulation of nucleolar heterochromatin formation during embryonic stem cell differentiation^33^ and interacts with XIST RNA^11,12^. Validation of the CIZ1-DHX9 complex was performed in vitro and in vivo and identified that DHX9 contributes to CIZ1 nucleolar localisation in S-phase. CIZ1 interacts with DHX9 in vitro and is localised to the nucleolus for approximately 4 h in early S-phase in HeLa cells. The nucleolus is a node for complex signalling networks that integrate cellular growth and protein synthesis by regulation of ribosome biogenesis. The localisation of DHX9 to the nucleolus can be inhibited by actinomycin D^48^ (Fig. 4A, D) and siRNA mediated depletion (Fig. 4C, G, H). Importantly both approaches prevent nucleolar recruitment of CIZ1, suggesting that DHX9 is required for recruitment of CIZ1 to the nucleolus during this window. The depletion of DHX9 appeared to specifically affect CIZ1 nucleolar recruitment as nucleolar localisation of the RNA polymerase I transcription factor UBF, and Nucleophosmin were unaffected by DHX9 depletion, consistent with normal nucleolar function in DHX9 depleted cells (Fig. 4).

CIZ1 is proposed to contribute to regulation of transcriptional networks in cancer cell lines^14,49^, regulation of DNA replication^5,6,8^ and contributes to XIST binding to the inactive X chromosome^11,12^. In each case, CIZ1 may serve as a molecular scaffold or recruitment factor to facilitate supramolecular assemblies. Of particular significance is recent observations that CIZ1 associates with XIST regulatory RNA at inactive X chromosomes^11,12^. Further analysis of the CIZ1 interactors identified here revealed 4 proteins (SRSF3, SRSF7, DDX5, and DDX17) that associate with XIST^32^, DHX9^50–52^ and CIZ1 (Table 1). The identification of common interactions from independent studies suggest that functional analysis of this is required to determine their potential roles in cell cycle regulation or XCI in future work.

The nucleolus is a highly dynamic structure that forms due to phase separation within the nucleus. The nucleolus integrates complex signals that integrate cellular growth, cell cycle phase and stress responses^53^. In addition to its canonical functions of rDNA transcription, chromosome dynamics and gene silencing and allelic exclusion, there are emerging non-canonical roles. Our data show that both DHX9 and CIZ1 shuttle into the nucleolus in a cell cycle phase regulated process. Protein shuttling from the nucleus to the nucleolus^54,55^ plays a key role in non-canonical nucleolar functions including telomere maintenance^56^, RNA pol II mediated transcription^57^, DNA repair^58,59^ and recombination^60^. Protein shuttling often involves nucleolar proteins that act as scaffolds for recruitment and include nucleolin and nucleophosmin. Importantly, both nucleophosmin and nucleolin were found to associate with CIZ1 in this study (Fig. 1C) and DHX9 is known to interact with nucleolin (Fig. 1D). In addition, nucleolar recruitment is associated with disordered structure^61^ and CIZ1 has a natively disordered N-terminal region that may contribute to its nucleolar shuttling. The data presented suggest that nucleolar CIZ1-DHX9 localisation is required for efficient cell cycle progression and contribute to non-canonical roles in the nucleolus.

The nucleolar recruitment of factors can contribute to regulation of the cell cycle^56^. The nucleolar recruitment of CIZ1 and DHX9 may contribute to accurate replication of the ribosomal DNA (rDNA). Both DHX9^34^ and Ciz1^5,6,8^ have been implicated in regulation of DNA replication via distinct mechanisms. rDNA represents a uniquely challenging template for DNA synthesis, which occurs at the nucleolar periphery during S-phase^36^ and requires temporospatial coordination with the transcriptional program. rDNA replication occurs in a bimodal program, with transcriptionally active regions replicating in early S-phase and inactive regions replicating in mid-late S-phase in NIH3T3 cells^62^. The perinucleolar region also appears to be a destination for the Xi chromosome in mid-late S-phase^63^, which engages in a CIZ1-dependent replication-linked transient visitation of the perinucleolar zone in normal primary mouse fibroblasts^13^. While this takes place within a ~ 30 min window in primary murine fibroblasts, the accumulation of CIZ1-DHX9 to these regions in early S-phase in Hela and BJ fibroblasts suggest that these events are unrelated to XCI, as this process is maintained in male and female cell lines. The recruitment of CIZ1-DHX9 to the nucleolus is coordinated and regulated within the cell cycle, suggesting that DHX9 and CIZ1 localisation to the nucleolus is required for efficient cell cycle progression. This is supported by observations that demonstrated that depletion of CIZ1 and DHX9 reduced cell cycle progression and S-phase entry^5,34^. The common roles for CIZ1 and DHX9 in epigenetic regulation may provide a mechanism for diverse roles of Ciz1 and DHX9 play in many biological processes. The data presented here have identified a network of proteins that are associated with nucleolar function and XCI. The data show Ciz1 interacts with DHX9 within the nucleolus for a short period of approximately 4 h at the beginning of S-phase. We propose that this transient nucleolar complex is required for efficient cell cycle progression, consistent with Ciz1 and DHX9 contributing to integration of cell cycle regulation and nucleolar function during early S-phase.

## Materials and methods

### Cell culture

HeLa cervical carcinoma epithelial cells, BJ hTERT-immortalized human fibroblasts and NIH3T3 murine fibroblasts were cultured in D-MEM (Gibco) low glucose, glutamax I, 10% FCS, penicillin, streptomycin and glutamine at 37 °C, 5% CO_2_/air in a humidified incubator. Cells were passaged by trypsinisation at 60% confluency and split 1 in 2 or 1 in 3. For cell synchrony experiments, 3T3 cells were passaged and seeded at 30–40% confluence, media changed 24 h later and cultured for a further 48 h until confluent. At confluence fresh media was applied and cells cultured for a further 72 h before releasing into fresh medium at lower density (1 in 4 dilution) to stimulate re-entry into the cell cycle. S-phase cells were produced using a double thymidine block, 24 h in 2.5 mM thymidine, released into fresh medium for 8 h before a second thymidine incubation for 16 h^5,64^. Cells were released into early S-phase by release into fresh medium for 1 h or 10 h for G2 cells, respectively. G2/M phase cells were produced by addition of 0.04 µg/ml nocodazole to S-phase cells for 12 h prior to isolation. G1 cells were produced as for G2/M synchronized cells, after which arrested cells were incubated for an additional 6 h in fresh media to complete mitosis and enter G1 phase. For immunofluorescence studies, cells were cultured on glass coverslips and synchronized as described above. To determine the number of cells in S-phase, EdU click it chemistry was used (Life Technologies) to label replicating nuclei. Percentage S-phase cells were determined by fluorescence microscopy scoring nuclei with replication foci as positive.

### Flow cytometry

Synchronised HeLa cells were produced by double thymidine treatment and released into fresh media. The cell cycle profile was determined by fixation overnight in 20% ethanol at − 20 °C and stained with 100 µg/ml propidium iodide in PBS, 0.5% v/v Triton X-100. Cell cycle profiles were determined using a BD FACScanto flow cytometer, and FACSDiva software. Data was collected for 10,000 cells with consistent gating applied for all samples.

### Nuclear extracts

Cells were washed in hypotonic buffer (10 mM HEPES, pH 7.8, 0.5 mM MgCl_2_, 5 mM K-acetate and 1 mM DTT), incubated in hypotonic buffer for 5 min and scrape harvested. Cell membranes were disrupted by dounce homogenisation (Wheaton) and nuclei isolated after centrifugation (10000* g*, 10 min) by removal of cytosolic proteins in supernatant. Nuclei were resuspended in hypotonic buffer, with the addition of 400 mM NaCl, and Complete EDTA free protease inhibitor cocktail (Roche). Nuclei were incubated for 5 min on ice and extracted proteins isolated by pelleting residual nuclei. The soluble fraction was used for binding studies with CIZ1 after correction of the NaCl concentration to 135 mM by dilution in hypotonic buffer.

### Protein expression and interaction studies

GST constructs were produced using the pGEX-6P3 vector containing full length ECIZ1, of derived fragments RD or N391 as described^5,8^. *E. coli* BL21 (DE3) were used to overexpress each protein using autoinduction media and culturing at 20 °C for 24 h. Proteins were purified as described^5^. After extensive washing of GST-CIZ1 coated beads, S-phase salt extracts were applied, bound for 1 h at 4 °C and washed extensively with 50 mM HEPES, 135 mM NaCl, 10 mM CaCl_2_, 20 mM MgCl_2_, 0.1% Triton X-100, 1 mM DTT and complete protease inhibitor cocktail (Roche). For analysis by western blot, proteins were eluted by boiling beads in SDS-PAGE loading buffer for 10 min, followed by electrophoresis through 4–15% precast TGX gels, and transfer to nitrocellulose (GE). For analysis by mass spectrometry, binding partners were eluted by overnight on-bead trypsinisation (Roche sequencing grade trypsin) diluted 1:50 in 100 mM sodium bicarbonate pH 8.0.

### Mass spectrometry

Peptides mixtures were loaded onto a polystyrene-divinylbenzene polymeric monolithic column (200 μm i.d. × 5 cm; LC Packings, NL) using an Ultimate nano-HPLC system (Dionex). Peptides were eluted at 3 μl/min flow rate over a 20 min linear gradient of aqueous 3–50% (v/v) acetonitrile containing 0.1% (v/v) heptafluorobutyric acid. Fractions were collected every 6 s onto a MALDI target plate using a probot microfraction collector (Dionex), followed by post-column 0.9 μl/min addition of 6 mg/ml α-cyano-4-hydroxycinnamic acid in aqueous 60% (v/v) acetonitrile^65^. Positive-ion MALDI mass spectra were acquired using an Applied Biosystems 4700 Proteomics Analyzer (Applied Biosystems, Foster City, CA, USA) in reflectron mode. MS spectra were acquired over between *m*/*z* 800–4,000. Monoisotopic masses were obtained from centroids of raw, unsmoothed data. CID fragmentation was performed on the 20 strongest precursors with a signal to noise (S/N) greater than 50 for each LC fraction, with a fraction-to-fraction precursor exclusion of 200 ppm applied. Collision energy of 1 kV was used, with air as the collision gas^66^. The precursor mass window was set to a relative resolution of 50, and the metastable suppressor was enabled. The default calibration was used for MS^2^ spectra, which were baseline-subtracted (peak width 50) and smoothed (Savitsky-Golay with three points across a peak and polynomial order 4); peak detection used a minimum S/N of 5, local noise window of 50 m*/z*, and minimum peak width of 2.9 bins^66^. Filters of S/N 20 and 30 were used for generating peak lists from MS and MS^2^ spectra, respectively. Peak lists were submitted to a locally-running copy of the Mascot search program (Matrix Science Ltd., version 2.1) via the Applied Biosystems GPS Explorer software interface^65,66^. Data were searched against the human subset of the NCBInr database. Search criteria specified: Enzyme, trypsin; fixed modifications, Methythio (C); Variable modifications, Oxidation (M); Peptide tolerance, 100 ppm; MS/MS tolerance, 0.1 Da; instrument, MALDI-TOF.TOF. Identification significance was determined by GPS using a total Ion score C.I. % threshold of 99.5%, and a minimum of two peptide matches.

### Antibodies for Western blotting and Immunofluorescence

Rabbit polyclonal antibodies were produced previously for ECIZ1, (anti-Ciz1 1793^6^), B23/nucleophosmin was purchased (sc-271737 Santa Cruz). Mouse monoclonal antibodies were used that recognise Actin (AC15), cyclin A (CY-1A) (Sigma Aldrich), UBF (sc-13125, Santa Cruz Biotech), DHX9 (sc-66997, Santa Cruz Biotech). Goat anti-rabbit peroxidase conjugates (Abcam), and goat anti-mouse peroxidase conjugates (Sigma Aldrich) were used with EtaC ECL Western blotting chemiluminescence reagent (Geneflow). Western blots were performed using 1% w/v BSA in TBS (Sigma Aldrich), 0.1% v/v Tween 20. For immunofluorescence, antibodies listed above were used at 1:100 dilution in PBS (Sigma Aldrich), 1% w/v BSA, 0.1% v/v, Triton X-100, 0.02% w/v SDS for 2 h at 37 °C, before extensive washing and labelling with fluorescent secondary antibodies (1:2000) for 1 h at 37 °C using either Goat anti mouse-Alexa Fluor 488 and Goat anti-rabbit Alexa Fluor 568 (Life Technologies/ThermoFisher). Cells were counter stained using DAPI- VectaShield to visualise nuclei.

### Nucleoli purification from S3 HeLa cells

Nucleolar purification was performed as described^44^, with minor modifications. Briefly, 10 × 15 cm dishes of HeLa cells at 80% confluence were used for isolation of purified nucleoli. Cells were was washed and incubated in Buffer A (10 mM HEPES pH 7.9, 1.5 mM MgCl_2_, 10 mM KCl, 0.5 mM DTT, 1 × Complete EDTA protease inhibitor tablet (Roche) for 5 min, scrape harvested and dounce homogenised. Nuclei were harvested by centrifugation at 228 × *g* for 5 min 4 °C. Nuclei were resuspended in S1 buffer (0.25 M Sucrose, 10 mM MgCl_2_) and layered over an equal volume of S2 (0.35 M Sucrose, 0.5 mM MgCl_2_) and nuclei pelleted by centrifugation at 1430×*g* for 5 min 4 °C. Purified nuclei were resuspended in buffer S2 and sonicated. Nuclei were examined by Differential interference contrast (DIC) microscopy and where intact nuclei were visible sonicated until nuclei had been effectively disrupted and nucleoli were visible. Nucleoli were isolated by layering the sonicated fraction over buffer S3 (0.88 M sucrose, 0.5 mM MgCl_2_) and centrifugation at 2800×*g* for 5 min 4 °C. DIC microscopy was used to confirm presence of nucleoli and loss of nuclei prior to analysis by western blotting.

### Immunoprecipitation reactions

HeLa cells were prepared and soluble nuclear extracts were prepared in binding buffer (50 mM HEPES pH 7.8, 10 mM MgCl_2_, 20 mM CaCl_2_ 10 mM magnesium acetate, 0.04% NP40, 2 × complete protease inhibitor cocktail (Roche), 10 mM ATP and 200 mM KCl). 2 μl of pre-immune rabbit sera or 2 μl of immunopurified N471 antibody (Covalabs^8^) were added as indicated for 2 h at 4 °C. Antibodies were immobilized on Immunopure protein A beads (Pierce Bioscience) for 1 h, washed five times (10 × bead volume) in a binding buffer diluted 2:3 with distilled water, and analysed by western blot.

### Transfection

Cells were transfected using Lonza nucleofection (Nucleofector 2C), program U-30 (NIH 3T3), I-013 (HeLa) or X-001 (BJ cells), as recommended. Cells were then plated in fresh media and cultured, either on coverslips for 24 h for investigation by immunofluorescence, or on dishes for later scrape harvesting in PBS (Sigma Aldrich), 1 × complete protease inhibitor (Roche), 1 mM DTT for analysis by western blotting. Transfection of synchronised populations of 3T3 cells was performed on G0 populations prior to release into cycle, using half a confluent 15 cm dish per reaction, with 5 μM Silencer Select siRNA (ThermoFisher). Silencer Negative Control No. 1 siRNA was used as a control transfection, with anti-DHX9 (siRNA ID: s4019, s4020 for HeLa and s231938 for mouse). 5 μM of each siRNA was used in 100 μL of Kit R transfection reagent (Lonza). Cells were plated into 3 × 9 cm plates for RNA extraction (PureLink RNA mini kit, ThermoFisher), EdU labelling to determine S-phase population of cells (ThermoFisher), and for protein extraction. Efficiency of transcript depletion was performed using quantitative real time PCR (qRT-PCR) using the ExPRESS superscript one-step qPCR kit (Life Technologies) and Taqman probes (Life technologies: murine cell lines (3T3) control GAPDH (Mm03302249_m1), CIZ1 (Mm00503766_m1) or DHX9 Mm00456021_m1 (3T3). Human specific Taqman probes Ciz1 (Hs00967155_m1) and DHX9 (Hs00357476_m1) and 18 s rRNA (Hs03003631_g1).

### Imaging

Images were taken using a Zeiss LSM 510 or LSM880 laser scanning confocal microscope at a scan speed 6, average of 4 frames sequentially for each fluorophore. Images were acquired with the pinhole set at 1 Airy unit for each wavelength used. Imaging with Zeiss LSM510 used HFT dichroic mirror set UV/488/543/633 used for excitation light and emission light collected with band pass (BP) 420–480 nm, BP 505–550 nm and long pass 560 nm. Imaging using a LSM880 used sequential imaging for minimal cross talk and collected light at 420–480 nm, 505–550 nm and 560–620 nm for DAPI, Alexa fluor 488 and Alexa Fluor 568 emissions respectively. Images were collected after optimisation of dynamic range to ensure sub-saturation. Colocalisation was determined with Image J colocalisation software, Coloc2, using the Costes method^67^. Images were cropped for presentation using Photoshop CS5.

### Statistical analysis

Significant differences in DNA replication activity were tested by one-way analysis of variance (ANOVA) followed by Tukey’s test *post-hoc* using IBM SPSS statistics 21. All significant results are shown in figures as appropriate where, **P < 0.01 and ***P < 0.001.

### STRING analysis

Protein interaction partners were analysed using STRING^68^ for CIZ1, ECIZ1-RD and shared CIZ1-DHX9 interaction partners. Functional interactions were produced and presented unaltered. Data uploaded to STRING will be made freely available on their website and local servers at Lancaster University.

### URLs

String: https://string-db.org

Biogrid: https://thebiogrid.org/

Coloc 2: https://imagej.net › Coloc_2.

## Supplementary information

Supplementary Video 1.

Supplementary Table and Figure Legends.

Supplementary Figures.

Supplementary Table 1.

Supplementary Table 2.

Supplementary Table 3.

Supplementary Table 4.

Supplementary Table 5.

Supplementary Table 6.
