# Supplementary material for: Identification of DHX9 as a cell cycle regulated nucleolar recruitment factor for CIZ1
Source: Sci Rep. 2020 Oct 22;10:18103. doi: 10.1038/s41598-020-75160-z (PMC7582970; doi:10.1038/s41598-020-75160-z)
Supplement: Supplementary file 2 — Supplementary Table and Figure Legends. [file 41598_2020_75160_MOESM2_ESM.docx]

**Supplementary tables**

**Table S1. Full dataset for ECIZ1 binding partners**. Identified interaction partners ranked by total ion score. Gene names for each interaction protein as used for STRING analysis, accession numbers, peptide count, confidence limits, total ion score, best ion score with C.I. % are shown. In addition, GO annotation, GO process, GO functional enrichment and KEGG pathways are shown in respective sheets within the spreadsheet.

**Table S2. Full dataset for ECIZ1-RD binding partners.** Identified interaction partners ranked by total ion score. Gene names for each interaction protein as used for STRING analysis, accession numbers, peptide count, confidence limits, total ion score, best ion score with C.I. % are shown. In addition, GO annotation, GO process, GO functional enrichment and KEGG pathways are shown in respective sheets within the spreadsheet.

**Table S3. Full dataset for ECIZ1-N391 binding partners.** Identified interaction partners ranked by total ion score. Gene names for each interaction protein as used for STRING analysis, accession numbers, peptide count, confidence limits, total ion score, best ion score with C.I. % are shown. In addition, GO annotation, GO process, GO functional enrichment and KEGG pathways are shown in respective sheets within the spreadsheet.

**Table S4. Full dataset for GST binding partners.** Identified interaction partners ranked by total ion score. Gene names for each interaction protein as used for STRING analysis, accession numbers, peptide count, confidence limits, total ion score, best ion score with C.I. % are shown. In addition, GO annotation, GO process, GO functional enrichment and KEGG pathways are shown in respective sheets within the spreadsheet.

**Table S5. ECIZ1 and RD mutual binding partners after negative selection.** Proteins were ranked by total ion score for CIZ1 dataset, with gene names and GO mapped gene terms shown. GO annotation, GO process, GO functional enrichment and KEGG pathways are shown in respective sheets within the spreadsheet.

**Table S6. ECIZ1 and DHX9 common interactors.** DHX9 binding partners were found in the BioGrid database and common interaction proteins with CIZ1 and DHX9 are shown here with GO annotation, GO process, GO functional enrichment and KEGG pathways are shown in respective sheets.

**Supplementary figures**

**Figure S1. STRING functional cluster analysis of ECIZ1 interactors.** ECIZ1 binding partners (Table S1) were analysed by STRING cluster analysis, which identified 3 clusters: Ribosomal (lower) RNA binding/Helicases (right) and nucleosomal (upper). Key identifies colour coding shows evidence of functional interactions.

**Figure S2. STRING functional cluster analysis of ECIZ1-RD interactors.** ECIZ1-RD interactors (Table S2) were analysed by STRING cluster analysis and identified 3 clusters: Ribosomal (central) RNA binding/Helicases (left) and nucleosomal (lower). Key shows colour coding for evidence of functional interactions.

**Figure S3. Full-length western blots for Figure 2.** A) Western blot of DHX9 for upper and lower panels Figure 2B. B) Western blots from Figure 2C showing CIZ1 load, DHX9 load and DHX9 immunoprecipitiation. C) Western blots from Figure 2D showing cyclin E and A levels (left), DHX9 (middle) and Actin (right). D) Western blots from Figure 2E showing DHX9 bands as indicated upper left, lower left, upper right and lower right panels respectively, as indicated. E) Western blots from Figure 2G showing DHX9 (left), CIZ1 (middle) and Actin (right). Boxes show selected bands for main figure.

**Figure S4. Z-stack reconstruction of CIZ1-DHX9 colocalisation.** HeLa cells were immunostained for CIZ1 (red), DHX9 (green) and DAPI used to stain DNA (Blue) in the merged image. Images shown are deconvoluted images acquired using DeltaVision fluorescence microscope. Black and white images are shown for highest contrast for DNA (DAPI), CIZ1 and DHX9 as indicated.

**Figure S5. Nucleolar colocalisation is CIZ1-DHX9 complex.** A. Confocal microscope images of **t**otal DNA (blue), DHX9 (green) and CIZ1 (red). In merged image, yellow indicates colocalisation. B. Analysis of colocalisation using FIJI colocalisation tool. DHX9 (green), CIZ1 (red) and colocalised pixels are shown in white. The correlation between pixels and intensity is shown in colocalised pixel map. Scale bar is 10 µm.

**Figure S6.** Full-length western blots for Figure 3D showing DHX9 (Left panel), CIZ1 (middle panel) and actin (right panel. Boxes show selected bands for main figure.

**Figure S7. Nucleolar colocalisation of CIZ1/DHX9 is dynamic and specific to early S-phase.** A) Flow cytometry profiles of propidium iodide stained DNA in HeLa cells representative of the cells shown in A. B) HeLa cells were synchronised at G2/M with thymidine/nocodazole treatments and CIZ1 and DHX9 visualised by confocal immunofluorescence microscopy. Time points refer to hours after release from nocodazole block. Total DNA is shown in blue; DHX9, green; CIZ1, Red and merged image, with yellow showing colocalisation. White bar = 10µm.

**Figure S8.** Full-length western blots for Figure 4D showing DHX9 (Left panel), CIZ1 (middle panel) actin (right panel and B23 nucleophosmin lower panel. Boxes show selected bands for main figure.

**Figure S9. Field images of** **HeLa cells after DHX9 siRNA treatment.** Mock, control siRNA, and DHX9 siRNA as indicated. Total DNA is blue, DHX9 is green and CIZ1 is red. In merged image yellow indicates colocalisation.

**Figure S10. Field images of** **HeLa cells after DHX9 siRNA treatment.** Mock, control siRNA, and DHX9 siRNA as indicated. Total DNA is blue, DHX9 is green and nucleophosmin (B23) is red. In merged image, yellow indicates colocalisation.
